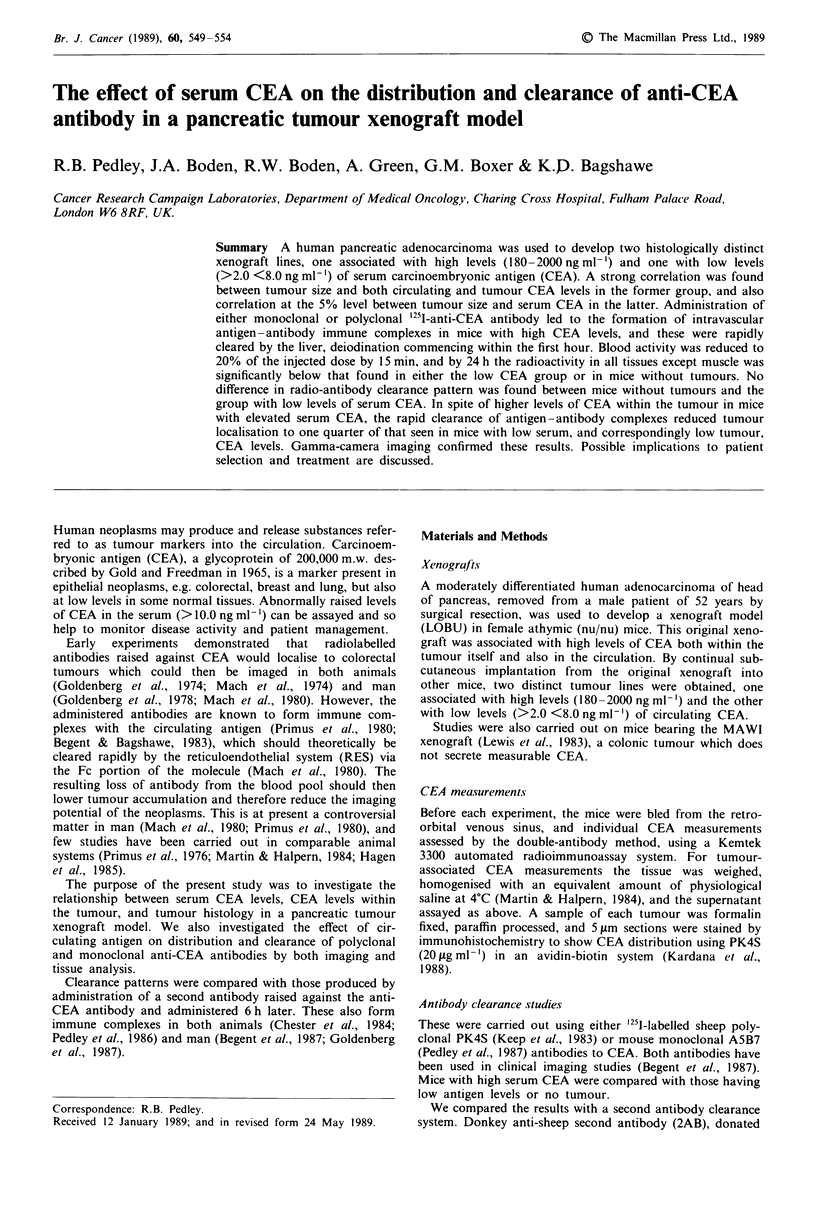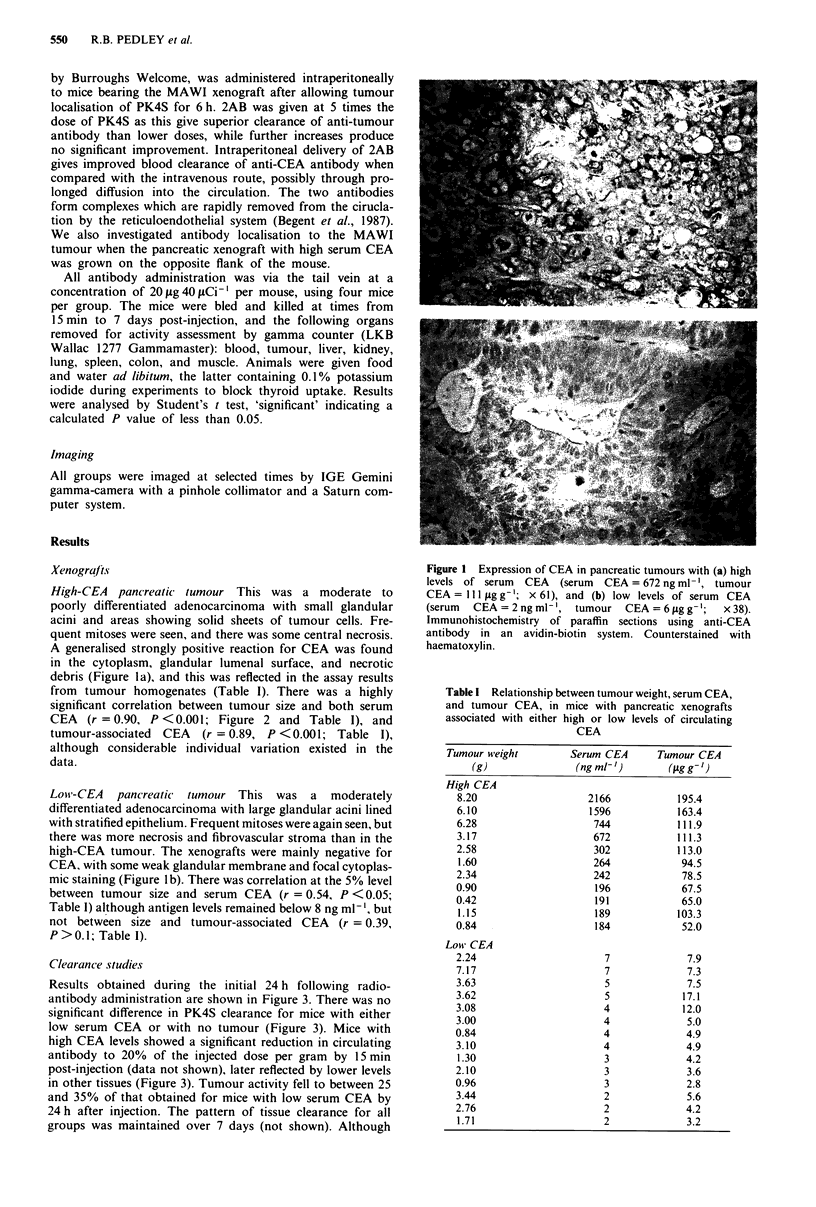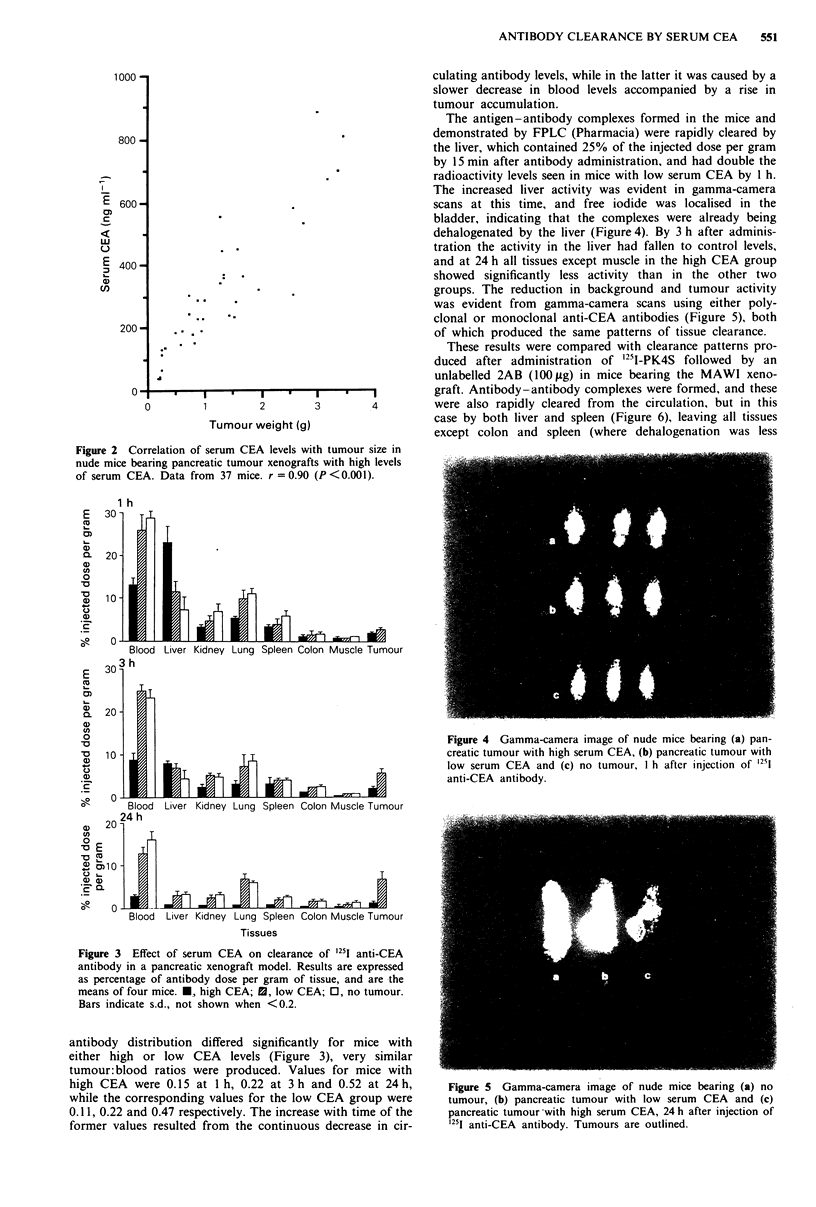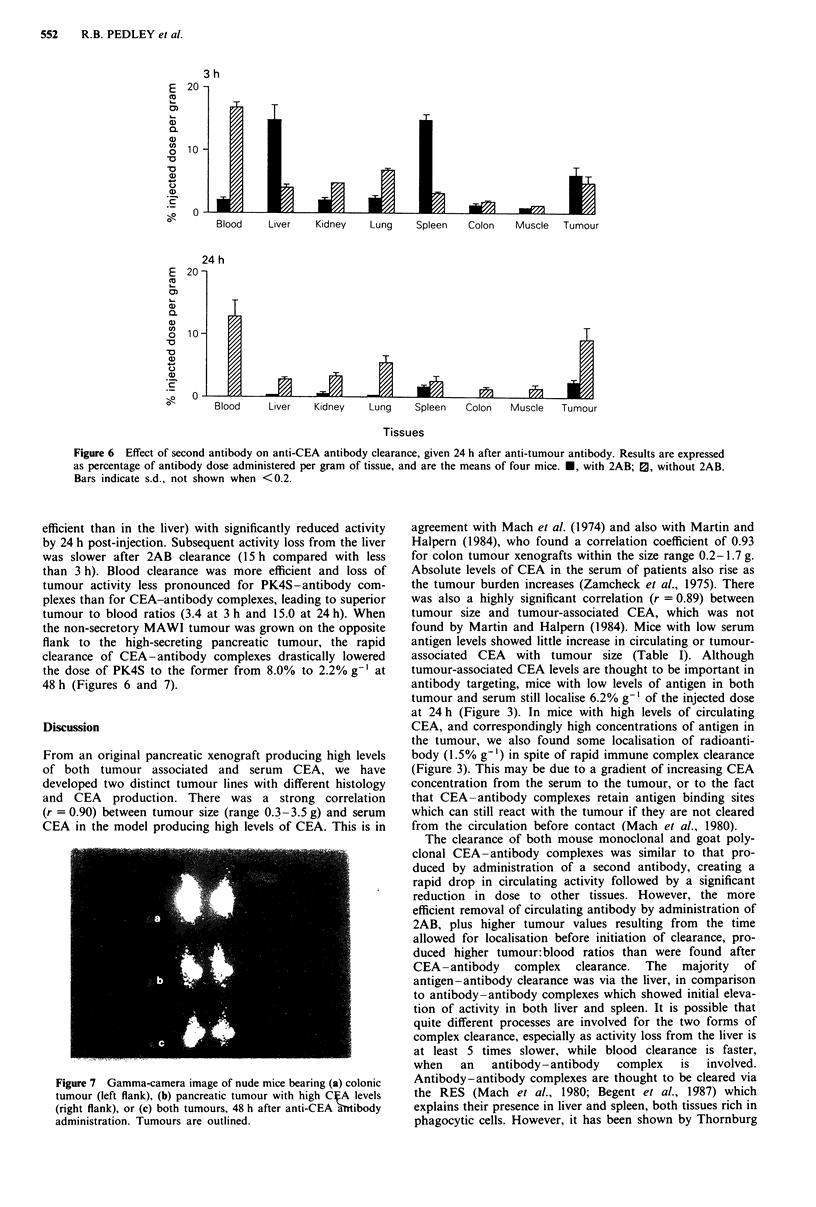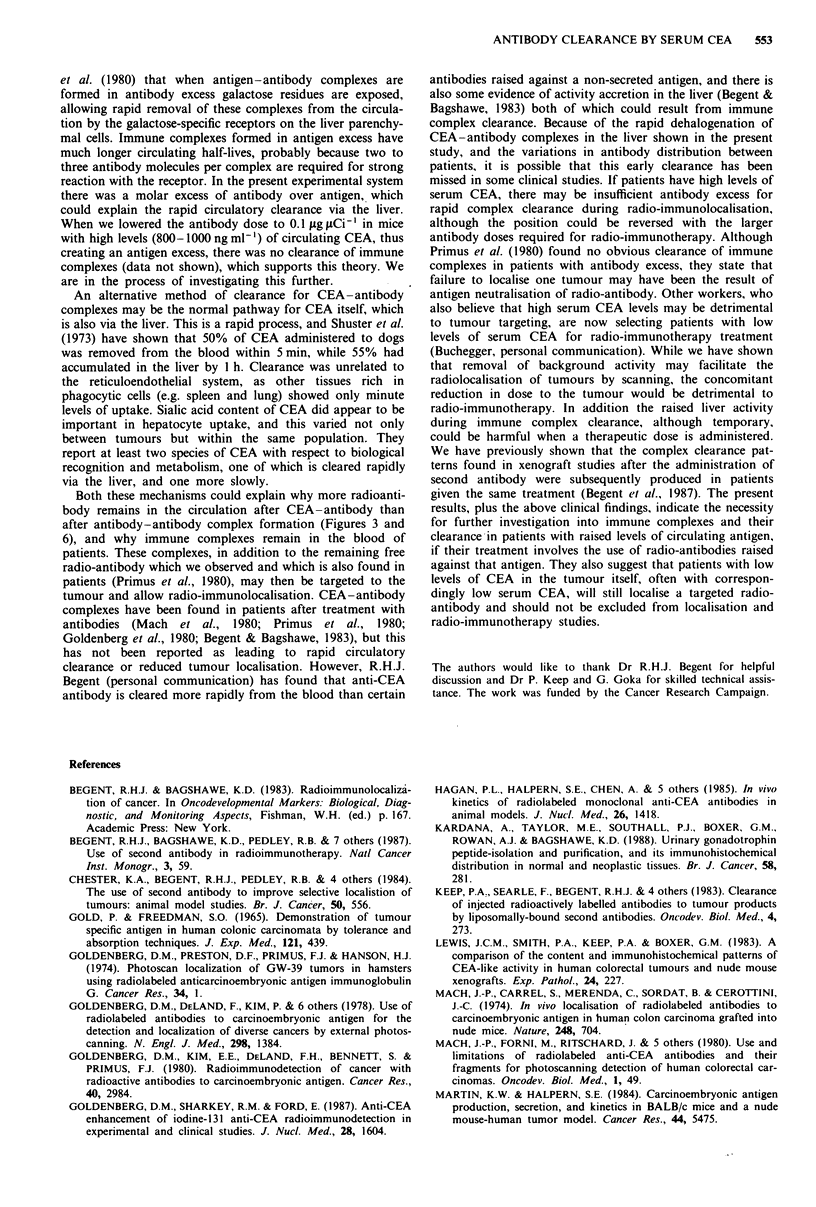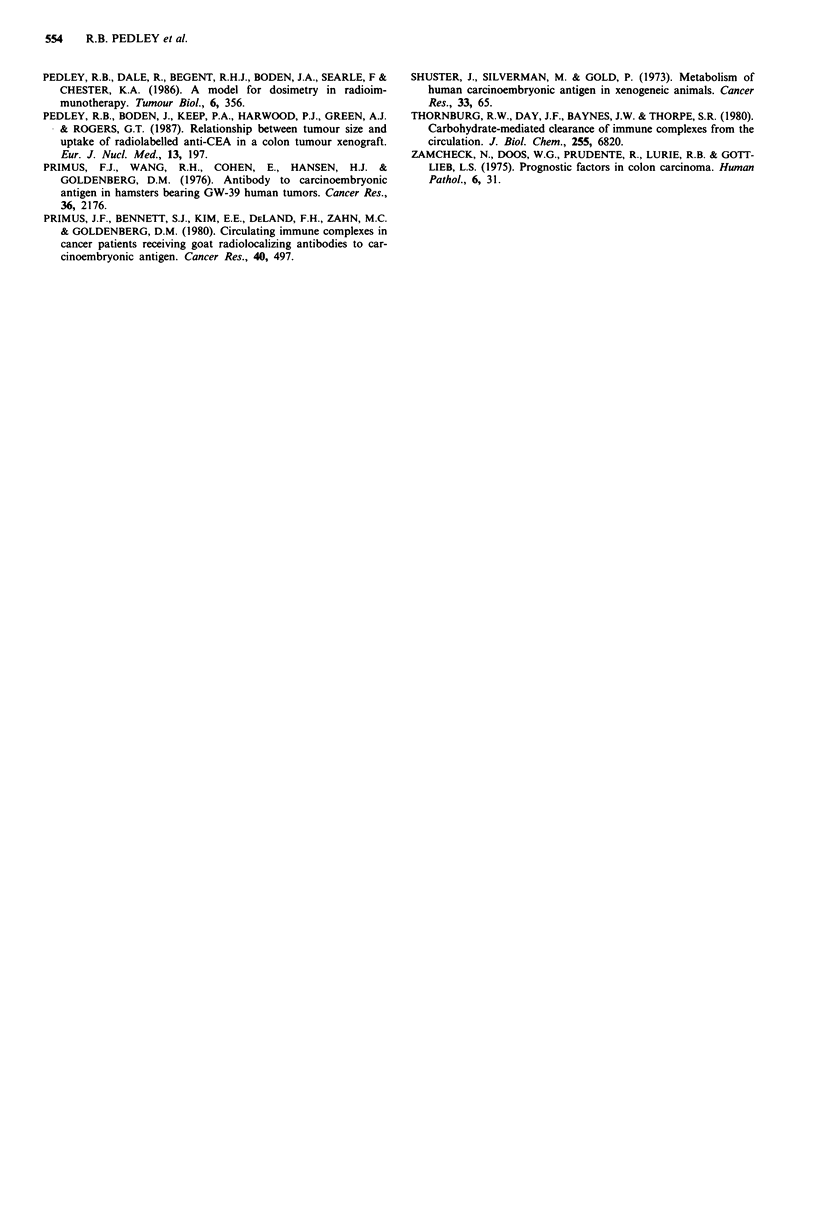# The effect of serum CEA on the distribution and clearance of anti-CEA antibody in a pancreatic tumour xenograft model.

**DOI:** 10.1038/bjc.1989.311

**Published:** 1989-10

**Authors:** R. B. Pedley, J. A. Boden, R. W. Boden, A. Green, G. M. Boxer, K. D. Bagshawe

**Affiliations:** Department of Medical Oncology, Charing Cross Hospital, London, UK.

## Abstract

**Images:**


Br. J. Cancer (1989), 60, 549-554
© The Macmillan Press Ltd., 1989

### The effect of serum CEA on the distribution and clearance of anti-CEA antibody in a pancreatic tumour xenograft model

R.B. Pedley, J.A. Boden, R.W. Boden, A. Green, G.M. Boxer & K.D. Bagshawe

*Cancer Research Campaign Laboratories, Department of Medical Oncology, Charing Cross Hospital, Fulham Palace Road, London W6 8RF, UK.*

**Summary** A human pancreatic adenocarcinoma was used to develop two histologically distinct xenograft lines, one associated with high levels (180–2000 ng ml$^{-1}$) and one with low levels (>2.0 <8.0 ng ml$^{-1}$) of serum carcinoembryonic antigen (CEA). A strong correlation was found between tumour size and both circulating and tumour CEA levels in the former group, and also correlation at the 5% level between tumour size and serum CEA in the latter. Administration of either monoclonal or polyclonal $^{125}$I-anti-CEA antibody led to the formation of intravascular antigen–antibody immune complexes in mice with high CEA levels, and these were rapidly cleared by the liver, deiodination commencing within the first hour. Blood activity was reduced to 20% of the injected dose by 15 min, and by 24 h the radioactivity in all tissues except muscle was significantly below that found in either the low CEA group or in mice without tumours. No difference in radio-antibody clearance pattern was found between mice without tumours and the group with low levels of serum CEA. In spite of higher levels of CEA within the tumour in mice with elevated serum CEA, the rapid clearance of antigen–antibody complexes reduced tumour localisation to one quarter of that seen in mice with low serum, and correspondingly low tumour, CEA levels. Gamma-camera imaging confirmed these results. Possible implications to patient selection and treatment are discussed.

Human neoplasms may produce and release substances referred to as tumour markers into the circulation. Carcinoembryonic antigen (CEA), a glycoprotein of 200,000 m.w. described by Gold and Freedman in 1965, is a marker present in epithelial neoplasms, e.g. colorectal, breast and lung, but also at low levels in some normal tissues. Abnormally raised levels of CEA in the serum (>10.0 ng ml$^{-1}$) can be assayed and so help to monitor disease activity and patient management.

Early experiments demonstrated that radiolabelled antibodies raised against CEA would localise to colorectal tumours which could then be imaged in both animals (Goldenberg *et al.*, 1974; Mach *et al.*, 1974) and man (Goldenberg *et al.*, 1978; Mach *et al.*, 1980). However, the administered antibodies are known to form immune complexes with the circulating antigen (Primus *et al.*, 1980; Begent & Bagshawe, 1983), which should theoretically be cleared rapidly by the reticuloendothelial system (RES) via the Fc portion of the molecule (Mach *et al.*, 1980). The resulting loss of antibody from the blood pool should then lower tumour accumulation and therefore reduce the imaging potential of the neoplasms. This is at present a controversial matter in man (Mach *et al.*, 1980; Primus *et al.*, 1980), and few studies have been carried out in comparable animal systems (Primus *et al.*, 1976; Martin & Halpern, 1984; Hagen *et al.*, 1985).

The purpose of the present study was to investigate the relationship between serum CEA levels, CEA levels within the tumour, and tumour histology in a pancreatic tumour xenograft model. We also investigated the effect of circulating antigen on distribution and clearance of polyclonal and monoclonal anti-CEA antibodies by both imaging and tissue analysis.

Clearance patterns were compared with those produced by administration of a second antibody raised against the anti-CEA antibody and administered 6 h later. These also form immune complexes in both animals (Chester *et al.*, 1984; Pedley *et al.*, 1986) and man (Begent *et al.*, 1987; Goldenberg *et al.*, 1987).

Correspondence: R.B. Pedley.
Received 12 January 1989; and in revised form 24 May 1989.

### Materials and Methods

### *Xenografts*

A moderately differentiated human adenocarcinoma of head of pancreas, removed from a male patient of 52 years by surgical resection, was used to develop a xenograft model (LOBU) in female athymic (nu/nu) mice. This original xenograft was associated with high levels of CEA both within the tumour itself and also in the circulation. By continual subcutaneous implantation from the original xenograft into other mice, two distinct tumour lines were obtained, one associated with high levels (180–2000 ng ml$^{-1}$) and the other with low levels (>2.0 <8.0 ng ml$^{-1}$) of circulating CEA.

Studies were also carried out on mice bearing the MAWI xenograft (Lewis *et al.*, 1983), a colonic tumour which does not secrete measurable CEA.

### *CEA measurements*

Before each experiment, the mice were bled from the retro-orbital venous sinus, and individual CEA measurements assessed by the double-antibody method, using a Kemtek 3300 automated radioimmunoassay system. For tumour-associated CEA measurements the tissue was weighed, homogenised with an equivalent amount of physiological saline at 4°C (Martin & Halpern, 1984), and the supernatant assayed as above. A sample of each tumour was formalin fixed, paraffin processed, and 5 $\mu$m sections were stained by immunohistochemistry to show CEA distribution using PK4S (20 $\mu$g ml$^{-1}$) in an avidin-biotin system (Kardana *et al.*, 1988).

### *Antibody clearance studies*

These were carried out using either $^{125}$I-labelled sheep polyclonal PK4S (Keep *et al.*, 1983) or mouse monoclonal A5B7 (Pedley *et al.*, 1987) antibodies to CEA. Both antibodies have been used in clinical imaging studies (Begent *et al.*, 1987). Mice with high serum CEA were compared with those having low antigen levels or no tumour.

We compared the results with a second antibody clearance system. Donkey anti-sheep second antibody (2AB), donated by Burroughs Welcome, was administered intraperitoneally to mice bearing the MAWI xenograft after allowing tumour localisation of PK4S for 6 h. 2AB was given at 5 times the dose of PK4S as this give superior clearance of anti-tumour antibody than lower doses, while further increases produce no significant improvement. Intraperitoneal delivery of 2AB gives improved blood clearance of anti-CEA antibody when compared with the intravenous route, possibly through prolonged diffusion into the circulation. The two antibodies form complexes which are rapidly removed from the circulation by the reticuloendothelial system (Begent *et al.*, 1987). We also investigated antibody localisation to the MAWI tumour when the pancreatic xenograft with high serum CEA was grown on the opposite flank of the mouse.

All antibody administration was via the tail vein at a concentration of 20 μg 40 $\mu Ci^{-1}$ per mouse, using four mice per group. The mice were bled and killed at times from 15 min to 7 days post-injection, and the following organs removed for activity assessment by gamma counter (LKB Wallac 1277 Gammamaster): blood, tumour, liver, kidney, lung, spleen, colon, and muscle. Animals were given food and water *ad libitum*, the latter containing 0.1% potassium iodide during experiments to block thyroid uptake. Results were analysed by Student's *t* test, 'significant' indicating a calculated *P* value of less than 0.05.

### *Imaging*

All groups were imaged at selected times by IGE Gemini gamma-camera with a pinhole collimator and a Saturn computer system.

### Results

### *Xenografts*

*High-CEA pancreatic tumour* This was a moderate to poorly differentiated adenocarcinoma with small glandular acini and areas showing solid sheets of tumour cells. Frequent mitoses were seen, and there was some central necrosis. A generalised strongly positive reaction for CEA was found in the cytoplasm, glandular lumenal surface, and necrotic debris (Figure 1a), and this was reflected in the assay results from tumour homogenates (Table I). There was a highly significant correlation between tumour size and both serum CEA ($r = 0.90$, $P < 0.001$; Figure 2 and Table I), and tumour-associated CEA ($r = 0.89$, $P < 0.001$; Table I), although considerable individual variation existed in the data.

*Low-CEA pancreatic tumour* This was a moderately differentiated adenocarcinoma with large glandular acini lined with stratified epithelium. Frequent mitoses were again seen, but there was more necrosis and fibrovascular stroma than in the high-CEA tumour. The xenografts were mainly negative for CEA, with some weak glandular membrane and focal cytoplasmic staining (Figure 1b). There was correlation at the 5% level between tumour size and serum CEA ($r = 0.54$, $P < 0.05$; Table I) although antigen levels remained below 8 ng $ml^{-1}$, but not between size and tumour-associated CEA ($r = 0.39$, $P > 0.1$; Table I).

### *Clearance studies*

Results obtained during the initial 24 h following radioantibody administration are shown in Figure 3. There was no significant difference in PK4S clearance for mice with either low serum CEA or with no tumour (Figure 3). Mice with high CEA levels showed a significant reduction in circulating antibody to 20% of the injected dose per gram by 15 min post-injection (data not shown), later reflected by lower levels in other tissues (Figure 3). Tumour activity fell to between 25 and 35% of that obtained for mice with low serum CEA by 24 h after injection. The pattern of tissue clearance for all groups was maintained over 7 days (not shown). Although

**Figure 1** Expression of CEA in pancreatic tumours with (**a**) high levels of serum CEA (serum CEA = 672 ng $ml^{-1}$, tumour CEA = 111 μg $g^{-1}$; ×61), and (**b**) low levels of serum CEA (serum CEA = 2 ng $ml^{-1}$, tumour CEA = 6 μg $g^{-1}$; ×38). Immunohistochemistry of paraffin sections using anti-CEA antibody in an avidin-biotin system. Counterstained with haematoxylin.

**Table I** Relationship between tumour weight, serum CEA, and tumour CEA, in mice with pancreatic xenografts associated with either high or low levels of circulating CEA

| *Tumour weight (g)* | *Serum CEA (ng $ml^{-1}$)* | *Tumour CEA (μg $g^{-1}$)* |
|---|---|---|
| *High CEA* | | |
| 8.20 | 2166 | 195.4 |
| 6.10 | 1596 | 163.4 |
| 6.28 | 744 | 111.9 |
| 3.17 | 672 | 111.3 |
| 2.58 | 302 | 113.0 |
| 1.60 | 264 | 94.5 |
| 2.34 | 242 | 78.5 |
| 0.90 | 196 | 67.5 |
| 0.42 | 191 | 65.0 |
| 1.15 | 189 | 103.3 |
| 0.84 | 184 | 52.0 |
| *Low CEA* | | |
| 2.24 | 7 | 7.9 |
| 7.17 | 7 | 7.3 |
| 3.63 | 5 | 7.5 |
| 3.62 | 5 | 17.1 |
| 3.08 | 4 | 12.0 |
| 3.00 | 4 | 5.0 |
| 0.84 | 4 | 4.9 |
| 3.10 | 4 | 4.9 |
| 1.30 | 3 | 4.2 |
| 2.10 | 3 | 3.6 |
| 0.96 | 3 | 2.8 |
| 3.44 | 2 | 5.6 |
| 2.76 | 2 | 4.2 |
| 1.71 | 2 | 3.2 |

**Figure 2** Correlation of serum CEA levels with tumour size in nude mice bearing pancreatic tumour xenografts with high levels of serum CEA. Data from 37 mice. $r = 0.90$ ($P < 0.001$).

**Figure 3** Effect of serum CEA on clearance of [125]I anti-CEA antibody in a pancreatic xenograft model. Results are expressed as percentage of antibody dose per gram of tissue, and are the means of four mice. ■, high CEA; ▨, low CEA; □, no tumour. Bars indicate s.d., not shown when <0.2.

antibody distribution differed significantly for mice with either high or low CEA levels (Figure 3), very similar tumour:blood ratios were produced. Values for mice with high CEA were 0.15 at 1 h, 0.22 at 3 h and 0.52 at 24 h, while the corresponding values for the low CEA group were 0.11, 0.22 and 0.47 respectively. The increase with time of the former values resulted from the continuous decrease in circulating antibody levels, while in the latter it was caused by a slower decrease in blood levels accompanied by a rise in tumour accumulation.

The antigen–antibody complexes formed in the mice and demonstrated by FPLC (Pharmacia) were rapidly cleared by the liver, which contained 25% of the injected dose per gram by 15 min after antibody administration, and had double the radioactivity levels seen in mice with low serum CEA by 1 h. The increased liver activity was evident in gamma-camera scans at this time, and free iodide was localised in the bladder, indicating that the complexes were already being dehalogenated by the liver (Figure 4). By 3 h after administration the activity in the liver had fallen to control levels, and at 24 h all tissues except muscle in the high CEA group showed significantly less activity than in the other two groups. The reduction in background and tumour activity was evident from gamma-camera scans using either polyclonal or monoclonal anti-CEA antibodies (Figure 5), both of which produced the same patterns of tissue clearance.

These results were compared with clearance patterns produced after administration of [125]I-PK4S followed by an unlabelled 2AB (100 µg) in mice bearing the MAWI xenograft. Antibody–antibody complexes were formed, and these were also rapidly cleared from the circulation, but in this case by both liver and spleen (Figure 6), leaving all tissues except colon and spleen (where dehalogenation was less

**Figure 4** Gamma-camera image of nude mice bearing (**a**) pancreatic tumour with high serum CEA, (**b**) pancreatic tumour with low serum CEA and (**c**) no tumour, 1 h after injection of [125]I anti-CEA antibody.

**Figure 5** Gamma-camera image of nude mice bearing (**a**) no tumour, (**b**) pancreatic tumour with low serum CEA and (**c**) pancreatic tumour with high serum CEA, 24 h after injection of [125]I anti-CEA antibody. Tumours are outlined.

**Figure 6** Effect of second antibody on anti-CEA antibody clearance, given 24 h after anti-tumour antibody. Results are expressed as percentage of antibody dose administered per gram of tissue, and are the means of four mice. ■, with 2AB; ▨, without 2AB. Bars indicate s.d., not shown when <0.2.

efficient than in the liver) with significantly reduced activity by 24 h post-injection. Subsequent activity loss from the liver was slower after 2AB clearance (15 h compared with less than 3 h). Blood clearance was more efficient and loss of tumour activity less pronounced for PK4S–antibody complexes than for CEA–antibody complexes, leading to superior tumour to blood ratios (3.4 at 3 h and 15.0 at 24 h). When the non-secretory MAWI tumour was grown on the opposite flank to the high-secreting pancreatic tumour, the rapid clearance of CEA–antibody complexes drastically lowered the dose of PK4S to the former from 8.0% to 2.2% $g^{-1}$ at 48 h (Figures 6 and 7).

### Discussion

From an original pancreatic xenograft producing high levels of both tumour associated and serum CEA, we have developed two distinct tumour lines with different histology and CEA production. There was a strong correlation ($r = 0.90$) between tumour size (range 0.3–3.5 g) and serum CEA in the model producing high levels of CEA. This is in agreement with Mach et al. (1974) and also with Martin and Halpern (1984), who found a correlation coefficient of 0.93 for colon tumour xenografts within the size range 0.2–1.7 g. Absolute levels of CEA in the serum of patients also rise as the tumour burden increases (Zamcheck et al., 1975). There was also a highly significant correlation ($r = 0.89$) between tumour size and tumour-associated CEA, which was not found by Martin and Halpern (1984). Mice with low serum antigen levels showed little increase in circulating or tumour-associated CEA with tumour size (Table I). Although tumour-associated CEA levels are thought to be important in antibody targeting, mice with low levels of antigen in both tumour and serum still localise 6.2% $g^{-1}$ of the injected dose at 24 h (Figure 3). In mice with high levels of circulating CEA, and correspondingly high concentrations of antigen in the tumour, we also found some localisation of radioantibody (1.5% $g^{-1}$) in spite of rapid immune complex clearance (Figure 3). This may be due to a gradient of increasing CEA concentration from the serum to the tumour, or to the fact that CEA–antibody complexes retain antigen binding sites which can still react with the tumour if they are not cleared from the circulation before contact (Mach et al., 1980).

**Figure 7** Gamma-camera image of nude mice bearing (**a**) colonic tumour (left flank), (**b**) pancreatic tumour with high CEA levels (right flank), or (**c**) both tumours, 48 h after anti-CEA antibody administration. Tumours are outlined.

The clearance of both mouse monoclonal and goat polyclonal CEA–antibody complexes was similar to that produced by administration of a second antibody, creating a rapid drop in circulating activity followed by a significant reduction in dose to other tissues. However, the more efficient removal of circulating antibody by administration of 2AB, plus higher tumour values resulting from the time allowed for localisation before initiation of clearance, produced higher tumour:blood ratios than were found after CEA–antibody complex clearance. The majority of antigen–antibody clearance was via the liver, in comparison to antibody–antibody complexes which showed initial elevation of activity in both liver and spleen. It is possible that quite different processes are involved for the two forms of complex clearance, especially as activity loss from the liver is at least 5 times slower, while blood clearance is faster, when an antibody–antibody complex is involved. Antibody–antibody complexes are thought to be cleared via the RES (Mach et al., 1980; Begent et al., 1987) which explains their presence in liver and spleen, both tissues rich in phagocytic cells. However, it has been shown by Thornburg

*et al.* (1980) that when antigen–antibody complexes are formed in antibody excess galactose residues are exposed, allowing rapid removal of these complexes from the circulation by the galactose-specific receptors on the liver parenchymal cells. Immune complexes formed in antigen excess have much longer circulating half-lives, probably because two to three antibody molecules per complex are required for strong reaction with the receptor. In the present experimental system there was a molar excess of antibody over antigen, which could explain the rapid circulatory clearance via the liver. When we lowered the antibody dose to 0.1 $\mu g \, \mu Ci^{-1}$ in mice with high levels (800–1000 $ng \, ml^{-1}$) of circulating CEA, thus creating an antigen excess, there was no clearance of immune complexes (data not shown), which supports this theory. We are in the process of investigating this further.

An alternative method of clearance for CEA–antibody complexes may be the normal pathway for CEA itself, which is also via the liver. This is a rapid process, and Shuster *et al.* (1973) have shown that 50% of CEA administered to dogs was removed from the blood within 5 min, while 55% had accumulated in the liver by 1 h. Clearance was unrelated to the reticuloendothelial system, as other tissues rich in phagocytic cells (e.g. spleen and lung) showed only minute levels of uptake. Sialic acid content of CEA did appear to be important in hepatocyte uptake, and this varied not only between tumours but within the same population. They report at least two species of CEA with respect to biological recognition and metabolism, one of which is cleared rapidly via the liver, and one more slowly.

Both these mechanisms could explain why more radioantibody remains in the circulation after CEA–antibody than after antibody–antibody complex formation (Figures 3 and 6), and why immune complexes remain in the blood of patients. These complexes, in addition to the remaining free radio-antibody which we observed and which is also found in patients (Primus *et al.*, 1980), may then be targeted to the tumour and allow radio-immunolocalisation. CEA–antibody complexes have been found in patients after treatment with antibodies (Mach *et al.*, 1980; Primus *et al.*, 1980; Goldenberg *et al.*, 1980; Begent & Bagshawe, 1983), but this has not been reported as leading to rapid circulatory clearance or reduced tumour localisation. However, R.H.J. Begent (personal communication) has found that anti-CEA antibody is cleared more rapidly from the blood than certain antibodies raised against a non-secreted antigen, and there is also some evidence of activity accretion in the liver (Begent & Bagshawe, 1983) both of which could result from immune complex clearance. Because of the rapid dehalogenation of CEA–antibody complexes in the liver shown in the present study, and the variations in antibody distribution between patients, it is possible that this early clearance has been missed in some clinical studies. If patients have high levels of serum CEA, there may be insufficient antibody excess for rapid complex clearance during radio-immunolocalisation, although the position could be reversed with the larger antibody doses required for radio-immunotherapy. Although Primus *et al.* (1980) found no obvious clearance of immune complexes in patients with antibody excess, they state that failure to localise one tumour may have been the result of antigen neutralisation of radio-antibody. Other workers, who also believe that high serum CEA levels may be detrimental to tumour targeting, are now selecting patients with low levels of serum CEA for radio-immunotherapy treatment (Buchegger, personal communication). While we have shown that removal of background activity may facilitate the radiolocalisation of tumours by scanning, the concomitant reduction in dose to the tumour would be detrimental to radio-immunotherapy. In addition the raised liver activity during immune complex clearance, although temporary, could be harmful when a therapeutic dose is administered. We have previously shown that the complex clearance patterns found in xenograft studies after the administration of second antibody were subsequently produced in patients given the same treatment (Begent *et al.*, 1987). The present results, plus the above clinical findings, indicate the necessity for further investigation into immune complexes and their clearance in patients with raised levels of circulating antigen, if their treatment involves the use of radio-antibodies raised against that antigen. They also suggest that patients with low levels of CEA in the tumour itself, often with correspondingly low serum CEA, will still localise a targeted radio-antibody and should not be excluded from localisation and radio-immunotherapy studies.

The authors would like to thank Dr R.H.J. Begent for helpful discussion and Dr P. Keep and G. Goka for skilled technical assistance. The work was funded by the Cancer Research Campaign.

### References

BEGENT, R.H.J. & BAGSHAWE, K.D. (1983). Radioimmunolocalization of cancer. In *Oncodevelopmental Markers: Biological, Diagnostic, and Monitoring Aspects*, Fishman, W.H. (ed.) p. 167. Academic Press: New York.

BEGENT, R.H.J., BAGSHAWE, K.D., PEDLEY, R.B. & 7 others (1987). Use of second antibody in radioimmunotherapy. *Natl Cancer Inst. Monogr.*, **3**, 59.

CHESTER, K.A., BEGENT, R.H.J., PEDLEY, R.B. & 4 others (1984). The use of second antibody to improve selective localistion of tumours: animal model studies. *Br. J. Cancer*, **50**, 556.

GOLD, P. & FREEDMAN, S.O. (1965). Demonstration of tumour specific antigen in human colonic carcinomata by tolerance and absorption techniques. *J. Exp. Med.*, **121**, 439.

GOLDENBERG, D.M., PRESTON, D.F., PRIMUS, F.J. & HANSON, H.J. (1974). Photoscan localization of GW-39 tumors in hamsters using radiolabeled anticarcinoembryonic antigen immunoglobulin G. *Cancer Res.*, **34**, 1.

GOLDENBERG, D.M., DELAND, F., KIM, P. & 6 others (1978). Use of radiolabeled antibodies to carcinoembryonic antigen for the detection and localization of diverse cancers by external photoscanning. *N. Engl. J. Med.*, **298**, 1384.

GOLDENBERG, D.M., KIM, E.E., DELAND, F.H., BENNETT, S. & PRIMUS, F.J. (1980). Radioimmunodetection of cancer with radioactive antibodies to carcinoembryonic antigen. *Cancer Res.*, **40**, 2984.

GOLDENBERG, D.M., SHARKEY, R.M. & FORD, E. (1987). Anti-CEA enhancement of iodine-131 anti-CEA radioimmunodetection in experimental and clinical studies. *J. Nucl. Med.*, **28**, 1604.

HAGAN, P.L., HALPERN, S.E., CHEN, A. & 5 others (1985). *In vivo* kinetics of radiolabeled monoclonal anti-CEA antibodies in animal models. *J. Nucl. Med.*, **26**, 1418.

KARDANA, A., TAYLOR, M.E., SOUTHALL, P.J., BOXER, G.M., ROWAN, A.J. & BAGSHAWE, K.D. (1988). Urinary gonadotrophin peptide-isolation and purification, and its immunohistochemical distribution in normal and neoplastic tissues. *Br. J. Cancer*, **58**, 281.

KEEP, P.A., SEARLE, F., BEGENT, R.H.J. & 4 others (1983). Clearance of injected radioactively labelled antibodies to tumour products by liposomally-bound second antibodies. *Oncodev. Biol. Med.*, **4**, 273.

LEWIS, J.C.M., SMITH, P.A., KEEP, P.A. & BOXER, G.M. (1983). A comparison of the content and immunohistochemical patterns of CEA-like activity in human colorectal tumours and nude mouse xenografts. *Exp. Pathol.*, **24**, 227.

MACH, J.-P., CARREL, S., MERENDA, C., SORDAT, B. & CEROTTINI, J.-C. (1974). *In vivo* localisation of radiolabeled antibodies to carcinoembryonic antigen in human colon carcinoma grafted into nude mice. *Nature*, **248**, 704.

MACH, J.-P., FORNI, M., RITSCHARD, J. & 5 others (1980). Use and limitations of radiolabeled anti-CEA antibodies and their fragments for photoscanning detection of human colorectal carcinomas. *Oncodev. Biol. Med.*, **1**, 49.

MARTIN, K.W. & HALPERN, S.E. (1984). Carcinoembryonic antigen production, secretion, and kinetics in BALB/c mice and a nude mouse-human tumor model. *Cancer Res.*, **44**, 5475.

PEDLEY, R.B., DALE, R., BEGENT, R.H.J., BODEN, J.A., SEARLE, F & CHESTER, K.A. (1986). A model for dosimetry in radioimmunotherapy. *Tumour Biol.*, **6**, 356.

PEDLEY, R.B., BODEN, J., KEEP, P.A., HARWOOD, P.J., GREEN, A.J. & ROGERS, G.T. (1987). Relationship between tumour size and uptake of radiolabelled anti-CEA in a colon tumour xenograft. *Eur. J. Nucl. Med.*, **13**, 197.

PRIMUS, F.J., WANG, R.H., COHEN, E., HANSEN, H.J. & GOLDENBERG, D.M. (1976). Antibody to carcinoembryonic antigen in hamsters bearing GW-39 human tumors. *Cancer Res.*, **36**, 2176.

PRIMUS, J.F., BENNETT, S.J., KIM, E.E., DELAND, F.H., ZAHN, M.C. & GOLDENBERG, D.M. (1980). Circulating immune complexes in cancer patients receiving goat radiolocalizing antibodies to carcinoembryonic antigen. *Cancer Res.*, **40**, 497.

SHUSTER, J., SILVERMAN, M. & GOLD, P. (1973). Metabolism of human carcinoembryonic antigen in xenogeneic animals. *Cancer Res.*, **33**, 65.

THORNBURG, R.W., DAY, J.F., BAYNES, J.W. & THORPE, S.R. (1980). Carbohydrate-mediated clearance of immune complexes from the circulation. *J. Biol. Chem.*, **255**, 6820.

ZAMCHECK, N., DOOS, W.G., PRUDENTE, R., LURIE, R.B. & GOTTLIEB, L.S. (1975). Prognostic factors in colon carcinoma. *Human Pathol.*, **6**, 31.